# Here and now: Yoga in Israeli schools

**DOI:** 10.4103/0973-6131.72629

**Published:** 2010

**Authors:** Miron Ehud, Bar-Dov An, Strulov Avshalom

**Affiliations:** 1 School of Public Health, Faculty of Social Welfare and Health Sciences, Mount Carmel, Haifa 31905; 2 District of Safed, Ministry of Health, Israel

**Keywords:** Postwar, school children, stress

## Abstract

**Context:**

In the aftermath of the Second Lebanon War, a project was initiated and designed to reduce tension in the children living in the area under bombardment.

**Aims:**

To assess the impact of yoga intervention in a group of Israeli school children residing in the region affected by the Second Lebanon War.

**Settings and Design:**

The study population included 122 school children aged 8–12 years in two elementary schools in Safed (*n*=55 and *n*=67, respectively) and their teachers (*n*=6). The children attended the third grade (*n*=28), fourth grade (*n*=42) and sixth grade (*n*=52).

Inclusion in the study was based on the school principal’s consent to participate in the program.

**Materials and Methods:**

Assessment was conducted using three questionnaires that have been previously validated in international studies and translated to Hebrew.

**Statistical Analysis Used:**

Statistical analysis of the results included Wilcoxon Signed Ranked Tests for pre- and post-intervention comparisons and the Kruskall–Wallis test for teacher and child cross-comparisons.

**Results:**

Based on the questionnaires completed by the children and their teachers, we found that the teachers reported many statistically significant improvements in the children’s concentration, mood and ability to function under pressure, although the children themselves were unaware of any change in their behavior. Enjoyment was reported by all participants, and almost all expressed an interest in continuing to practice yoga during school hours. We conclude that participation in yoga classes may be both enjoyable and beneficial to children living in stressful conditions.

**Conclusions:**

The study indicates that yoga may be beneficial as an intervention for children in postwar stress situations.

## INTRODUCTION

Yoga is an ancient method of spiritual practices that has evolved tremendously over several thousand years, branching into different schools, styles and philosophies. Yoga typically consists of a combination of body postures, breathing exercises and various mental and/or physical disciplines. When performed properly, the physical exercises (“asanas”) focus the person on the performance in that moment in time (“here and now”). In this way, the exercises direct the person’s concentration to the present and reduce anxiety about what may or may not happen in the future. The asanas themselves require no exotic equipment or clothing. They are simple to learn and many can be performed almost anywhere.[1,2] Proper performance of asanas rewards participants with a sense of achievement and competence.

Today, as in the past, many claims are made for yoga’s health-related benefits. A Google search for “Yoga and Relaxation” yields 2,360,000 citations. A search for “Yoga and Children and Relaxation” yields 1,890,000 citations. The vast majority of these citations are anecdotal reports of yoga experiences or directories of yoga classes. Although many studies have been conducted on yoga’s effects on adult practitioners, few systematic attempts have been made to study yoga’s effects on children. This is particularly surprising in light of yoga’s increasing popularity as an activity taught in schools.

### Medical effects of yoga

A systematic review of the professional literature found moderate evidence of the effectiveness of yoga (Viniyoga) in reducing chronic low-back pain.[3] As a result, in their joint clinical practice guideline, the American College of Physicians and the American Pain Society recommend to clinicians to consider the addition of nonpharmacologic therapy with proven benefits for chronic or subacute low-back pain for patients who do not improve with self-care options (weak recommendation, moderate-quality evidence).[4]

According to a second systematic review, yoga was significantly associated with pain reduction in studies of mind–body interventions for older adults with chronic nonmalignant pain.[5]

In a randomized controlled trial of yoga in the treatment of migraine without aura, a significant reduction in migraine headache frequency and associated clinical features in patients treated with yoga was reported over a period of 3 months.[6]

The effects of yoga and ayurveda on geriatric depression were evaluated in 69 individuals over the age of 60 years in a residential home. The depression symptom scores of the yoga group at both 3 and 6 months decreased significantly from a group average baseline of 10.6 to 8.1 and 6.7, respectively, while the control group showed no change over the same period.[7]

Yoga has been studied as a complementary treatment modality for diverse conditions, including pregnancy,[8,9] carpal tunnel syndrome,[10] asthma,[11] schizophrenia,[12] pancreatitis[13] and various cancers.[14,15]

A randomized controlled trial of yoga among 128 breast cancer patients suggests that yoga is associated with beneficial effects on social functioning, although low adherence was observed during the trial.[16]

Notably, there is very little high-grade evidence of the effectiveness of yoga in medical conditions. Most studies do not follow rigorous scientific standards, or are based on small samples, precluding the possibility of drawing generalized conclusions from their results.

### Yoga effects in children

Several studies have investigated the effects of yoga in children. In a randomized controlled trial, adolescents with inflammatory bowel disease undergoing a yoga intervention reported lower levels of functional disability, less use of emotion-focused avoidance and lower anxiety following the intervention compared with adolescents in the control group. When the pre- and postintervention data for the two groups were combined, adolescents had significantly lower scores for gastrointestinal symptoms and emotion-focused avoidance following the yoga intervention.[17]

The effects of yoga have also been examined in the treatment of eating disorders among 45 fifth-grade girls. The yoga intervention group showed a significant reduction in scores measuring body dissatisfaction and drive for thinness.[18]

Another study showed the beneficial effects of yoga on spatial memory scores in comparison with a fine-arts control group.[19]

### Yoga in schools

One of the few systematic studies on the effects of yoga in schoolchildren was conducted by Simeon P. Slovacek, Susan A. Tucker and Laura Pantoja. In 2003, they published “A Study of the Yoga Ed Program at the Accelerated School,” based on a study that they had conducted at one inner-city Los Angeles school. A total of 405 students in grades K-8 and 18 core subject and yoga teachers were involved in the study. The study reported that yoga class participation helped students improve their behavior, physical health and academic performance, and also improved students’ attitudes toward themselves.[20]

In Israel, yoga has been endorsed by the Ministry of Education and funded by the Karev Fund. As a result, yoga classes are taught in many schools throughout Israel during school hours to children aged 5–18 years. Additionally, yoga classes are held after school hours in community centers or privately run businesses throughout the country. These classes seem to be very popular.

### Posttraumatic stress effects and yoga

The use of yoga in treating posttraumatic stress disorder (PTSD) has been described in the psychiatric literature and recommended as an effective adjunct to psychiatric therapy in depression and PTSD.[21]

Yoga has been assessed in several PTSD groups, including Vietnam-war veterans,[21] tsunami survivors and case reports from 9/11 survivors.[22]

In high-school students exposed to war in the Kosovo area, mind–body skills were shown to reduce the effects of PTSD in repeated measurements of the posttraumatic stress score.[23]

## OBJECTIVES

To assess the impact of yoga intervention in a group of Israeli school children residing in the region affected by the Second Lebanon War.

## MATERIALS AND METHODS

The program “Here and Now: Yoga in School” was developed immediately after the Second Lebanon War. The war left in its wake many thousands of children, residents of Northern Israel, whose basic sense of well-being and security had been shattered by the shelling of their homes. The majority of these children come from economically disadvantaged families that cannot afford private psychological treatment. Local government authorities have limited financial resources to help these children regain their sense of well-being.

As a result of her own experiences in this war, the developer of the program “Here and Now” believed that asana performance might alleviate anxiety and stress. The program was designed to reach Israeli schoolchildren who otherwise would have no access to long-term therapeutic modalities.

The study population included 122 school children aged 8–12 years in two elementary schools in Safed (*n*=55 and *n*=67, respectively) and their teachers (*n*=6). The children attended the third grade (*n*=28), fourth grade (*n*=42) and sixth grade (*n*=52).

Inclusion in the study was based on the school principal’s consent to participate in the program.

Three questionnaires were distributed to the study participants:

1. A questionnaire based on the WHO (Five) Well-Being Index (1998)[24] was distributed to the children before and after the intervention.
2. A questionnaire based on the Conners Abbreviated Symptom Questionnaire[25] was distributed to the teachers before and after the intervention.
3. A satisfaction questionnaire was distributed to the children after the intervention.

The intervention comprised 13 yoga training sessions conducted over a period of 4 months. Sessions were incorporated into the regular school schedule rather than as a special after-school activity. The sessions were led by trained yoga teachers with experience both in the practice of yoga and in work with children.

A list of Asanas and Pranayama is provided in Appendix A.

Statistical analysis of the results included Wilcoxon Signed Ranked Tests for pre- and post-intervention comparisons and the Kruskall–Wallis test for teacher and child cross-comparisons.

## RESULTS

The Conners Abbreviated Symptom Questionnaire is a 10-item tool for external observer assessment of child behavior. Observers are asked to rate the frequency of the following observed behaviors:

1. Restless and overactive
2. Excitable, impulsive
3. Disturbs other children
4. Fails to finish things he/she starts – short attention span
5. Constantly fidgeting
6. Inattentive, easily distracted
7. Demands must be met immediately – easily frustrated
8. Cries easily and often
9. Mood changes quickly and drastically
10. Temper outbursts, explosive and unpredictable behavior

Results were first analyzed for the intervention group as a whole, which showed a statistically significant improvement in attention span, restlessness and inattentiveness, on comparing the pre- and post-intervention measures [Table 1].

**Table 1:** Summary of statistically significant differences in behavior

Behavior	z-test statistics*	Asymp. sig. (2-tailed)
Fails to finish things he/she starts – short attention span	-2.251	0.024
Constantly fidgeting	-3.342	0.001
Inattentive, easily distracted	-2.128	0.033

* Based on positive ranks

Improvements were also indicated in all remaining items, with the exception of disturbing other children, although the change was not statistically significant.

In the second stage of data analysis, the results were classified by grade – third grade (N_pre_=N_post_=28), fourth grade (N_pre_=42, N_post_=37) and sixth grade (N_pre_=52, N_post_=51) and analyzed by mean–rank (Kruskall–Wallis test).

The pre- and post-intervention assessment differences were statistically significant (*P*<0.05) for all three grade groups [Table 2]. While the third and sixth graders showed an improvement in the observed behavior symptoms after the intervention, fourth graders showed a decline in the observed behavioral symptoms. The reason for the discrepancy may well be the interobserver variation of the three teachers who observed the children before and after the intervention.

**Table 2:** Statistically significant differences in behavior by age group

Behavior	Mean difference (after-before)		
	3^rd^ grade	4^th^ grade	5^th^ grade
Demands must be met immediately–easily frustrated	-0.357	0.233	-0.088
Cries easily and often	-0.107	0.274	-0.004
Mood changes quickly and drastically	-0.071	0.062	-0.048
Temper outbursts, explosive and unpredictable behavior	-0.179	0.230	-0.023

The WHO (Five) Well-Being Index (1998) measures the absence of a positive effect and has been validated for use in children. The index measures the following five items, which are rated on a six-point Likert-like scale, with reference to the preceding 2-week period:

1. I have felt cheerful and in good spirits.
2. I have felt calm and relaxed.
3. I have felt active and vigorous.
4. I woke up feeling fresh and rested.
5. My daily life has been filled with things that interest me.

Analysis indicated that the children experienced no differences in these items, comparing pre- and post-intervention scores.

We analyzed the satisfaction questionnaire and found that children expressed satisfaction with the yoga training they had received. The children found the yoga classes interesting (57%) and fun (64%) and wished to continue them as part of the school schedule (90%).

Raw data tables are provided in Appendix B–Appendix C for independent analysis.

## DISCUSSION

The reported study combines several unique features that facilitate the analysis of the effects of yoga on stress reduction. Specifically, the study incorporated an intervention for children who had experienced a period of intensive stress, where the yoga intervention was conducted shortly after the occurrence of the stress-inducing events.

Another unique feature of this study is the use of standardized questionnaires for the effects of yoga, combining external observation and self-assessment reports.

The study findings are in accordance with previous evidence on the benefit of yoga in PTSD 22 and specifically, contribute to the assessment of yoga in children who may be suffering from PTSD.

The limitations of the study include the absence of a control group of children in the region, who had similarly experienced high stress as a result of the Second Lebanon War. This limitation is shared by similar studies.[23]

Another concern is inconsistency between self-assessment and external-observer results. This may be explained by the young age of the respondents and their age-limited ability for self-observation.

This issue warrants further studies but nonetheless indicates a potentially positive role and effect of yoga in the management of children in poststress situations.

## Appendix A

### Pranayama

Because the participants of the study were children (aged 8–12 years), we did not emphasize formal breathing exercises that included timing inhalation and exhalation and/or placement of breath in various parts of the body. This is in accordance with the recommendations of Geeta Iyenga (“Yoga for Schoolchildren”, www.iyengar-yoga.org il, 7/06; Hebrew translation).

### Asanas

Due to the nature of the target population (Hebrew-speaking children between the ages of 8 and 12 years), the various asanas were referred to by their Hebrew equivalents, as listed in a very helpful resource book (Yoga For Children: Ruth Aharoni, Neora Publishers; in Hebrew). The following list contains the English translations of the names used for the asanas in the classes, grouped according to categories.

### Animals

Lion, cat, dog, snake (Cobra), camel, eagle, bird, fish, turtle, butterfly, crow, pigeon, rabbit, frog, cow’s head, stork, spider, grasshopper, lizard.

### People

Warrior, woodchopper, dancer, archer, embryo (child’s pose).

### Objects

Slide, plank, scythe, candle, bridge, boat, table, triangle, balloon, ball, swing, wheel, airplane.

### Nature

Tree, mountain, rainbow, crescent (moon).

### Other

Headstands, handstands, twists, forward and backward bends.

## Appendix B

### Participant satisfaction questionnaire

**Table:** What bid you especially enjoy at the yoga sessions?

Category	Frequency
I liked everything	36
I did not like anything	4
I liked doing the exercises (*asanas*)	3
I liked the balance exercises	3
“Sun salutations”	5
The calm atmosphere	6
“Lion” (*Simhasana*)	1
“Panther” (sic)	1
The yoga mats	2
The bell	3
The reduction in nerves	4
Tibetan bowls	21
“The Slide” (*Purvottanasana*)	2
“The planet Earth” (meditation/visualization)	1
We are excused from our regular lessons!	1
Missing	29
Total	122

**Table:** What would you like to change about the sessions?

Category	Frequency
Nothing (no changes necessary)	42
The general atmosphere	5
It should be less calm	1
We should learn more	4
I would change everything	3
The time (when the sessions take place)	3
“Sun salutations”	4
“Downward Dog” (*Adho Mukha Svanasana*)	1
Difficult exercises	1
The teacher should yell less!	2
We should have better mats	1
It should be less noisy	16
The Tibetan bowls should be louder	2
It should be more fun	2
Eye exercises	1
“Shalom” at end of session (variation of “OM”)	1
The bell	1
“Personal space” (not interfering with others)	1
Missing	31
Total	122

**Table:** Degree of interest generated

Category	Frequency
None	8
Very little	8
Some interest	25
Much interest	27
Very much	30
Missing	24
Total	122

**Table:** How enjoyable were the yoga sessions

Category	Frequency
Not at all	9
Very little	7
Some	18
Much	32
Very much	32
Missing	24
Total	122

**Table:** How practical were the Yoga sessions?

Category	Frequency
Not at all	17
Very little	14
Somewhat	13
Quite practical	30
Very practical	24
Missing	24
Total	122

**Table:** Would you like the Yoga sessions to continue as part of the school schedule?

Category	Frequency
Not at all	12
Very little	8
Some	9
Much	19
Very much	51
Missing	23
Total	122

**Table:** Did you feel relaxed after the Yoga sessions?

Category	Frequency
Not at all	14
Very little	13
Somewhat relaxed	14
Quite relaxed	20
Very relaxed	38
Missing	23
Total	122

## Appendix C

### Observed behavior of participants

**Table:** Observed behavior

Behavior	Class	Number of observations	Mean rank
Satisfaction	3	28	68.54
	4	37	46.74
	6	51	61.52
Mood change	3	28	63.29
	4	37	48.23
	6	51	63.32
Rage	3	28	61.77
	4	37	46.03
	6	51	65.75
Crying	3	28	59.82
	4	37	51.09
	6	51	63.15
Total		116	

**Table:** Observed behavior average values

Behavior	Satisfaction		Rage		Crying		Mood change	
class	Before	After	Before	After	Before	After	Before	After
3	0.5714	0.2143	0.3214	0.2143	0.2857	0.2143	0.3929	0.2143
4	0.5238	0.7568	0.4286	0.7027	0.2619	0.3243	0.5	0.7297
6	0.6393	0.5517	0.582	0.5776	0.4098	0.3621	0.5574	0.5345
